# Fetal Y chromosome abnormalities cause false-low fetal fraction in NIPT: a retrospective analysis of 24,101 pregnant women

**DOI:** 10.3389/fgene.2026.1856523

**Published:** 2026-06-10

**Authors:** Siqi Hu, Lu Zhou, Yong Xu, Weipeng Wu, Niping Jiang, Ying Hao, Hu Zhang, Yang Liu, Weiqing Wu, Bo Wu, Wenlan Liu

**Affiliations:** 1 The Center for Medical Genetics, Shenzhen Maternity and Child Healthcare Hospital, Women and Children’s Medical Center, Southern Medical University, Shenzhen, Guangdong Province, China; 2 Shenzhen Key Laboratory of Maternal and Child Health and Diseases, Shenzhen, Guangdong Province, China; 3 Shenzhen Key Laboratory of Birth Defect Prevention and Control, Shenzhen, Guangdong Province, China

**Keywords:** cell-free DNA, fetal fraction, FF-QuantSC, non-invasive prenatal testing (NIPT), sex chromosome abnormalities, y-chromosome abnormalities

## Abstract

**Background:**

Low fetal fraction is a common cause of NIPT no-call results, leading to repeat sampling and delayed clinical decisions. In cases of fetal sex chromosome abnormalities (SCAs), the standard Y chromosome-based method for estimating fetal fraction (FF) may be unreliable, potentially leading to misinterpretation of low-FF results.

**Methods:**

We retrospectively analyzed 24,101 pregnant women who underwent NIPT between January 2023 and January 2025. FF was estimated using the Y chromosome-based method for male fetuses and FF-QuantSC for female fetuses. Confirmatory invasive testing was performed for high-risk or failed cases.

**Results:**

The SCA prevalence was significantly higher in the low-FF group (FF<3.5%, n = 70) than in the adequate-FF group (FF ≥ 3.5%, n = 24,020), representing a ∼78-fold increased risk (11.43% versus 0.21%; OR = 78.227, *P* < 0.001). All eight SCAs in the low-FF group involved Y-chromosome abnormalities. Re-estimation using FF-QuantSC corrected the FF to a reportable level in all eight cases, confirming “false-low” FF, estimation using the Y chromosome-based method. Subsequent NIPT analysis, enabled by the qualified FF, successfully reported high-risk results for SCAs (45,X or Y deletions). Moreover, analysis of Y-chromosome read distribution profiles in these eight low-FF cases provided visual clues suggestive of these Y-chromosome abnormalities.

**Conclusion:**

Low FF in NIPT is strongly associated with fetal Y-chromosome abnormalities, suggesting that Y chromosome-based FF estimation may produce a “false-low” FF in some cases with fetal Y-chromosome abnormalities. We suggest that re-estimating FF using FF-QuantSC combined with Y-chromosome read distribution analysis may be considered before reporting test failure.

## Introduction

Since the discovery of cell-free fetal DNA in maternal plasma by Lo et al. (1997), non-invasive prenatal testing (NIPT), which detects fetal chromosomal abnormalities by analyzing cell-free DNA (cfDNA) in maternal plasma, has become a routine test in many countries (Jayashankar et al., 2023). NIPT has been confirmed as the most accurate screening test for trisomies 21, 18 and 13 (Zhang et al., 2015), demonstrating significantly lower false positive rates and higher detection rates than traditional serum screening (Gil et al., 2017). Accumulating evidence also supports its effectiveness in screening for sex chromosome abnormalities (SCAs) (Xu et al., 2019), copy number variations (CNVs) (Xiang and Peng 2021), and rare autosomal trisomies (RATs) (Xiang et al., 2023).

However, approximately 1% of initial NIPT samples fail to yield a result and are reported as “no-call” (Dungan et al., 2023). The most frequent cause of no-call results is low fetal fraction (FF), defined as the proportion of circulating cfDNA originating from the placenta relative to total maternal plasma cfDNA (Dungan et al., 2023). Critically, many studies have reported that low FF correlates with an increased risk of fetal chromosomal aneuploidy and adverse pregnancy outcomes (Revello et al., 2016; Chan et al., 2017; Chang et al., 2021). Samples with no-call results due to low FF typically require repeat sampling or invasive diagnosis, which delays turnaround time, increases healthcare costs, and heightens maternal anxiety. It is well established that FF is influenced by numerous factors, including maternal characteristics (e.g., high body mass index, low-molecular-weight heparin) (Rolnik et al., 2018; Burns et al., 2017), fetal-placental characteristics (e.g., gestational age) (Qiao et al., 2019b), and pregnancy-related factors (e.g., *in vitro* fertilization (IVF), twin gestation) (Lee et al., 2018; Hedriana et al., 2020). Of note, in addition to biological factors, experimental factors and specific FF calculation algorithms may also affect FF (Qiao et al., 2019a; Deng and Liu, 2022).

Accurate FF estimation is therefore critical, as FF serves as a gatekeeping quality metric for NIPT and directly impacts downstream clinical management. Laboratories use diverse algorithms for FF assessment (Cserhati, 2021). For male fetuses, the Y chromosome-based method is the most widely used and is considered the gold standard for FF estimation (Dennis Lo et al., 2008). However, emerging evidence suggests that fetal SCAs—particularly those involving the Y chromosome—may compromise Y chromosome-based FF estimation, potentially producing an apparently “false-low” FF. Research in this area remains limited (Zeng et al., 2020; Xie et al., 2024). Alternative approaches include single-nucleotide polymorphism (SNP)-based, cfDNA count-based, differential methylation-based, cfDNA size-based, and nucleosome track-based methods (Peng and Jiang 2017). Among these, the accurate Quantification of FF with Shallow-Coverage sequencing of maternal plasma DNA (FF-QuantSC) is a common method that employs a neural network model and utilizes differential genomic patterns between fetal and maternal genomes to quantify FF (Yuan et al., 2020). This method does not rely on sex chromosome information and is therefore applicable to both male and female fetuses, while also avoiding inaccuracies in FF measurement caused by SCAs. Despite these advances, clinical data validating the performance of such methods, particularly regarding associations between low FF and fetal SCAs, remain limited.

In this study, we retrospectively analyzed 24,101 pregnant women who underwent NIPT at our hospital between January 2023 and January 2025, aiming to investigate the association between NIPT failure due to low FF and fetal SCAs. Furthermore, we aimed to suggest possible clinical management approaches for low-FF results by employing a sex chromosomes-independent FF estimation method (FF-QuantSC) combined with Y-chromosome read distribution profiling.

## Methods

### Subjects

This retrospective study enrolled pregnant women (≥12 weeks’ gestation with singleton or twin pregnancy) who underwent NIPT at Shenzhen Maternity and Child Healthcare Hospital from January 2023 to January 2025. Following pretest counseling, written informed consent was obtained from all participants. Baseline characteristics including maternal age, gestational age, BMI, IVF, and pregnancy type (singleton or twin pregnancy) were recorded. The study protocol was approved by the Ethics Review Committee of the Shenzhen Maternity and Child Healthcare Hospital (Approval No. SFYLS [2024]130).

### Non-invasive prenatal testing

Five milliliters of maternal blood was collected from pregnancies in Cell-Free DNA Collection Tubes (GeenSeek, Guangzhou, China). Blood plasma was collected after centrifugation at 3,900 *g* for 15 min at 4 °C. cfDNA was extracted from 200 µL of plasma using a DNA Extraction Kit (BGI Biotech, Wuhan, China). Fetal Chromosome Aneuploidies (T21, T18, and T13) Detection Kit (Combinatorial Probe-Anchor Synthesis Sequencing Method, CPAS) (BGI Biotech, Wuhan, China) was used for library construction (involving end-repairing, A-base tailing and adaptor ligation, followed by additional amplification by PCR). The libraries were quantified using a Qubit 3.0 Fluorometer (LifeTech, Invitrogen, Carlsbad, CA, United States), and 48 libraries with different labels were equally pooled and quantified. After a double-stranded DNA library was constructed, DNA Nanoballs (DNBs) was generated by denaturation, circularization and rolling circle amplification (RCA). These DNBs were then quantified (8–40 ng/μL) and loaded onto sequencing chips, followed by sequencing on the MGISEQ-2000 sequencing platform (MGI, Shenzhen, China). All 35 bp sequencing reads were aligned to the human reference genome (hg19/GRCh37).

To calculate the FF, different methods were adopted for pregnancy with male or female fetuses. For male fetuses, the FF could be calculated by the proportion of reads mapping to Y-chromosome relative to those mapping to autosomal chromosomes (Y chromosome-based method). The FF in pregnancies with female fetuses was estimated using the FF-QuantSC algorithm developed by BGI as mentioned before (Yuan et al., 2020). A minimum threshold of FF was 3.5% (Zhang et al., 2015). Initial low FF cases were recommended for sample redraw after 2–4 weeks. After two failures, a test failure report was offered and confirmatory invasive testing was recommended. For samples that passed quality control, a binary hypothesis Z-score was applied to classify aneuploidy (Lau et al., 2012). Z-score was set within the range from −3 to 3 as the threshold to evaluate the risk of chromosomal aneuploidies. The original NIPT testing was performed prospectively as part of routine clinical diagnostics. For the purpose of this retrospective study, the existing sequencing data were re-analyzed, but no samples were re-sequenced.

### Confirmatory invasive testing

Pregnant women with test failure or high-risk NIPT results were recalled for genetic counseling and recommended for confirmatory invasive testing via amniocentesis, including karyotyping analysis and chromosome microarray analysis (CMA). All individuals made a voluntary decision about whether to undergo the test. Karyotyping was processed using a conventional G-banded method, CMA was performed by Affymetrix CytoScan 750 K arrays (Thermo Fisher Scientific Inc., Waltham, Massachusetts, United States) following the manufacturer’s protocols.

### Statistical analysis

Linear regression analysis was conducted to investigate the association between FF and gestational age. Multivariable logistic regression was performed to evaluate the independent association between low FF and fetal SCAs after adjusting for maternal age, gestational age, maternal BMI, IVF, and twin pregnancy. The results were reported as odds ratios (OR) with corresponding 95% confidence intervals (CI). Statistical analyses were performed with IBM SPSS 26.0 (IBM Corp, Armonk, NY), and *P* < 0.05 was considered statistically significant.

## Results

### Cohort overview

**FIGURE 1:**
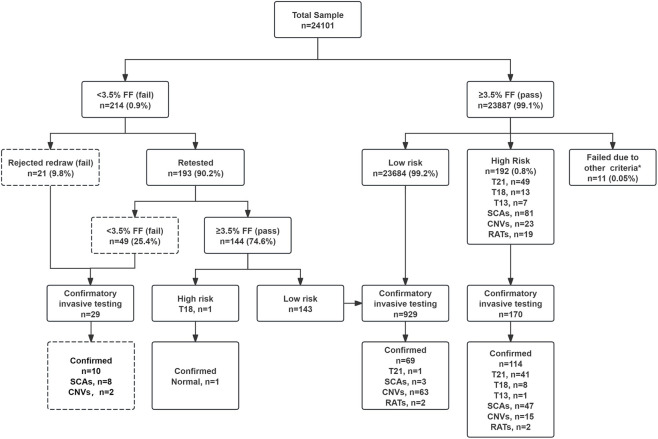
Flowchart of NIPT results. *Other criteria include a borderline Z score, defined as a Z score between 1.96 and 3 for chromosomes 13, 18, or 21, or the presence of multiple chromosomal aberrations (MCA, ≥4).

A total of 24,101 pregnant women underwent NIPT between January 2023 and January 2025. Initial test failure due to low FF (FF <3.5%) occurred in 214 cases (0.9%, Figure 1). Of these, 193 cases underwent sample redraw and repeat testing, while 21 rejected redraw, primarily due to previous test failures at another laboratory or advanced gestational age. The average interval between first sample draw and second sample draw was 3.1 weeks (SD = 1.54). After retesting, 144 (74.6%) achieved adequate FF (FF ≥ 3.5%), while 49 (25.4%) showed persistently low FF. Therefore, 70 cases (comprising 21 with no redraw and 49 with repeated low FF) constituted the low-FF group for subsequent analysis. Confirmatory invasive testing in this group confirmed 10 cases of fetal chromosomal abnormalities (8 SCAs and 2 CNVs). Meanwhile, among the 144 resolved cases, 143 were classified as low-risk. The remaining high-risk case (suspected T18) was later confirmed to have a normal karyotype by invasive testing.

Among the initial 23,887 cases with adequate FF, 11 (0.05%) failed to yield a reportable NIPT result due to persistent borderline Z score or multiple chromosomal aberrations (MCA). Of the remaining 23,876 cases with reportable results, 192 (0.8%) were identified as high-risk. Following genetic counseling, 170 of these high-risk cases underwent invasive testing, which confirmed 114 true positives: 41 T^21^, 8 T^18^, 1 T^13^, 47 SCAs, 15 CNVs, and 2 RATs. In addition, among the low-risk NIPT results, 929 cases voluntarily underwent invasive testing, which identified 69 chromosomal abnormalities, including 1 T^21^, 3 SCAs, 2 RATs, and 63 CNVs (mostly microdeletions or microduplications below the NIPT detection limit).

### Baseline characteristics

**FIGURE 2:**
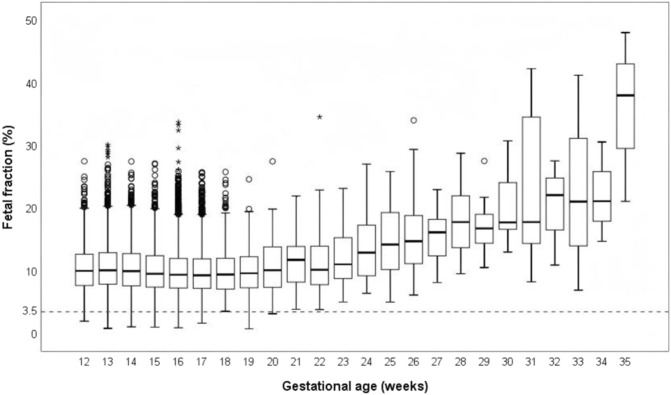
Associations between fetal fraction and gestational age. A box plot illustrates the relationship between fetal fraction and gestational age, based on 24,090 samples. The lower and upper boundaries of each box correspond to the 25th and 75th percentiles, respectively, with the central line indicating the median (50th percentile). The whiskers extend to the 5th and 95th percentiles. Outliers are represented by open circles, and asterisks denote extreme values. Linear regression analysis indicates a modest positive correlation between fetal fraction and gestational age in pregnancies beyond 20 weeks (*R* ^2^ = 0.27, *P* < 0.001). Dotted line indicates the 3.5% FF threshold.

Table 1 summarizes the baseline characteristics for all pregnancies (n = 24,090) included in the statistical analysis, using mean, standard deviation, median, quartiles and percentages by NIPT results (11 cases failed due to borderline Z score or MCA were not included in statistics). The low-FF (n = 70) and adequate-FF (n = 24,020) groups were comparable in gestational age at initial draw (mean: 15.84 versus 15.81 weeks) and maternal age (mean: 31.65 versus 31.65 years). As expected, the low-FF group had higher maternal BMI (mean: 24.25 versus 21.09 kg/m^2^) and higher proportions of IVF (30.0% versus 11.68%) and twin pregnancies (11.43% versus 2.92%) (P < 0.05). Among twin pregnancies, the proportion of IVF-conceived twins did not differ between the low-FF and adequate-FF groups (62.5% vs. 63.8%). Analysis of the entire cohort revealed a positive correlation between FF and gestational age. The median FF at 12th weeks was approximately 10% and remained relatively stable until 20th weeks. A modest positive correlation between FF and gestational age was observed after 20 weeks (R^2^ = 0.27, *P* < 0.001, Figure 2). Among 70 cases with low-FF, pregnancy outcomes were available for 55, with 49 live births and a preterm delivery rate of 26.5% (13/49). These data were presented in Supplementary Table S1.

**TABLE 1:** Baseline characteristics of cases included in statistical analysis (n = 24,090).

	NIPT result		
Baseline characteristics	Total* (n = 24,090)	FF<3.5% (n = 70)	FF ≥ 3.5% (n = 24,020)
Fetal fraction (%)			
Mean	10.19	2.42	10.30
SD	3.86	0.93	3.78
Median	9.67	2.77	9.73
Q1-Q3	7.45–12.39	1.37–3.22	7.54–12.44
Gestational age at initial draw (weeks)			
Mean	15.81	15.84	15.81
SD	2.02	2.22	2.02
Median	16.14	16.00	16.14
Q1-Q3	14.14–16.86	14.04–16.96	14.14–16.86
Maternal age (years)			
Mean	31.65	31.65	31.65
SD	3.98	4.63	3.98
Median	31.00	32.00	31.00
Q1-Q3	29–34	29–35	29–34
Maternal BMI (kg/m^2^)			
Mean	21.10	24.25	21.09
SD	2.89	4.89	2.87
Median	20.66	23.07	20.65
Q1-Q3	19.13–22.64	20.79–26.09	19.11–22.63
Pregnancy achieved via IVF			
Yes (%)	2828 (11.74%)	21 (30%)	2807 (11.69%)
No (%)	21,262 (88.26%)	49 (70%)	21,213 (88.31%)
Twin pregnancy			
Yes (%)	710 (2.95%)	8 (11.43%)	702 (2.92%)
No (%)	23,380 (97.05%)	62 (88.57%)	23,318 (97.08%)

* 11 cases failed due to borderline Z score or MCA, were not included in the statistic; SD, standard deviation. Q1-Q3, interquartile range (25th-75th percentiles).

### Association between low fetal fraction and fetal SCAs

Table 2 summarizes 10 cases with fetal chromosomal abnormalities in the low-FF group (n = 70) and their confirmatory invasive testing results. These included eight cases of SCAs (cases 1–8) and two cases of autosomal CNVs (cases 9–10). Table 3 lists the 50 cases of SCAs ultimately confirmed in the adequate-FF group (n = 24,020), including 47 true positives and 3 false negatives. The prevalence of SCAs differed markedly between groups. In the low-FF group, 11.43% (8/70) of cases were confirmed as SCAs, compared to only 0.21% (50/24,020) in the adequate-FF group. Multivariable logistic regression analysis, adjusting for maternal age, gestational age, maternal BMI, IVF, and twin pregnancy, demonstrated that low FF was significantly associated with fetal SCAs (OR = 78.227, 95% CI: 34.563–177.054, P < 0.001). Strikingly, all 8 confirmed SCAs in the low-FF group involved structural abnormalities or mosaicism loss of the Y-chromosome.

**TABLE 2:** 10 cases with fetal chromosomal abnormalities in the low-FF group (FF<3.5%).

Case	NIPT	CMA	Karyotype
1	Fail	arr [GRCh37]Yp11.32q11.21 (118,551_14,568,324)x2 Yq11.21q11.23 (14,568,325_28,799,654)x0	mos 45,X [28]/46,X,psu idic(Y)(q11.21)[132]
2*	Fail	arr (X)x1,(Y)x0.64	—
3	Fail	arr (X)x1,(Y)x2	46,X,psu idic(Y)(q12)
4	Fail	arr [GRCh37] Xp22.33 or Yp11.32p11.31 (168,552_2,690,819 or 118,552_2,640,819)x3, Yp11.31q11.221 (2,650,425_18,274,478)x2, Yq11.221q11.23 (18481645_28,799,654)x0	mos 46,X,psu idic(Y)(q11.221)[46]/45,X [14]/46,XY [4]
5	Fail	arr (X)x1,(Y)x0.85	mos 45,X [5]/46,XY [51]
6	Fail	arr [GRCh37] Yp11.32q11.223 (118,552_23,660,178)x0.7, Yq11.223q11.23 (23683718_28,799,654)x0	mos 45,X [13]/46,X,del(Y)(q11.222)[30]
7*	Fail	^a^arr [GRCh37] Yp11.32q11.221 (118,552_19,556,683)x2 [0.6], Yq11.222q11.23 (20618888_28,799,654)x0	^a^mos 45,X [19]/46,X,psu idic(Y)(q11.22)[22]
		^b^arr [GRCh37] Yp11.32q11.221 (118,552_19,567,327)x1 [0.3], Yq11.221q11.23 (19563600_28,799,654)x0	^b^mos 45,X [41]/46,X,psu idic(Y)(q11.22)[15]/46,X,del(Y)(q11.22)[8]
8	Fail	arr (X,N)×1, (1–22)×2	mos 45,X [10]/46,XY [50]
9	Fail	arr [GRCh37] 16p11.2 (29351827_30,350,748)x1	46,XY
10	Fail	arr [GRCh37] 4q11q12 (52696792_58,320,842)x1	46,XY

* Case 2 rejected the chromosome karyotype analysis. Case 7 was monochorionic diamniotic twin pregnancy, “a” and “b” indicate “fetus a” and “fetus b”, respectively.

**TABLE 3:** 50 cases with fetal sex chromosomal abnormalities in the adequate-FF group (FF ≥ 3.5%).

Case	NIPT	CMA	Karyotype
1	45,X	arr (X)x1 [0.13]	mos 45,X [50]/47,XXX [6]
2	45,X	arr [GRCh37] Yp11.32q11.223 (118,552_24,017,592)x1.5	mos 47,X,psu idic(Y)(q11.22),+mar mat [67]/47,X,del(Y)(q11.22),+mar mat [3]
3	45,X	arr (X)x1,(Y)x0.69	mos 45,X [20]/46,XY [30]
4	45,X	arr [GRCh37] Xq21.2q28 (85762588_155,233,098)x1	46,X,del(X)(q21.2)
5	45,X	arr [GRCh37]Xp22.33q21.33 (168,552_96,897,921)x1.8, Xq21.33q28 (96918479_155,233,098)x1	mos 46,X,del(X)(q21.3)[37]/45,X [12]/47,X,psu idic(X)(q21.3),+3 [4]
6	45,X	arr (X)x1,(Y)x0.5	mos 45,x [29]/47,xyy [2]/46,xy [51]
7	45,X	arr [GRCh37] Yq11.223q11.23 (24070172_28,799,654)x0	mos 45,X [40]/46,X,psu idic(Y)(q11.21)[22]/46,X,del(Y)(q11.21)[18]
8	45,X	arr (X)x1 [0.48]	mos 45,X [42]/47,XXX [8]
9	45,X	arr [GRCh37] Xp22.33p11.4 (168,552_40541753)x1	46,X,del(X)(p11.4)
10–11	45,X	arr (X)x3	47,XXX
12–24	47,XXX	arr (X)x3	47,XXX
25	47,XXY	arr (X)x2,(Y)x1	mos 47,XXY [41]/46,XY [9]
26–41	47,XXY	arr (X)x2,(Y)x1	47,XXY
42–47	47,XYY	arr (X)x1,(Y)x2	47,XYY
48	Low-risk	arr (X)x3	47,XXX
49	Low-risk	arr (X)x1 [0.22]	mos 45,X [20]/46,XX [30]
50	Low-risk	arr (X)x3 [0.2]	mos 47,XXX [5]/46,XX [80]

Cases 1–47 are true positives (NIPT, high-risk), and cases 48–50 are false negatives (NIPT, low-risk).

Regarding the two autosomal CNV cases in the low-FF group (Case 9: 16p11.2 deletion; Case 10: 4q11q12 deletion), the absence of recurrent CNVs in the adequate-FF group and lack of statistical significance suggest these likely represent stochastic events rather than low FF-associated findings.

### Re-estimation of fetal fraction using FF-QuantSC

Table 4 summarizes the FF values of 10 fetal chromosomal abnormalities cases in the low-FF group measured by the Y chromosome-based method and FF-QuantSC, respectively. The FF estimated based on the Y-chromosome in the first and second sample draw of these 10 cases were both below the threshold (<3.5%). After recalculating FF using the FF-QuantSC algorithm, all 8 fetal SCAs cases (cases 1–8) exhibited qualified FF levels (range: 5.375%–19.897%), demonstrating that the initial low FF represented a “false-low” FF measurement with Y chromosome-based method. Subsequent NIPT analysis based on the corrected FF values accurately reported high risk for SCAs (45,X or Y deletions). In contrast, the two autosomal CNV cases (cases 9–10) remained below the FF threshold after FF-QuantSC recalculation, confirming a true low FF status.

**TABLE 4:** Re-estimation of fetal fraction using FF-QuantSC in 10 low fetal fraction cases.

Case	FF (Initial)	FF (Redraw)	NIPT result (Y-based method)	FF (FF-QuantSC)	NIPT result (FF-QuantSC)
1	2.674	2.425	Fail	11.382	45,X; del (Yq11.21-q11.223)
2	1.053	—	Fail	5.375	45,X
3	1.213	1.067	Fail	10.878	45,X; del (Yq11.21-q11.223)
4	1.372	0.919	Fail	8.755	45,X
5	1.018	—	Fail	14.524	45,X; del (Yp11.2-q11.223)
6	1.068	1.305	Fail	6.326	45,X
7	1.923	—	Fail	19.897	45,X; del (Yp11.2-q11.23)
8	1.972	3.04	Fail	5.8	45,X
9	2.272	2.46	Fail	2.963	Fail
10	3.404	3.205	Fail	3.177	Fail

FF (initial) and FF (redraw) refer to the fetal fraction estimated by the Y chromosome-based method for the first and second draw, respectively. Case 2, case 5 and case 7 rejected the second sample draw. FF (FF-QuantSC) refer to the fetal fraction recalculated by the FF-QuantSC, method. FF ≥ 3.5% passed quality control.

Using confirmatory invasive testing as the reference, the sensitivity, specificity, PPV and NPV for SCA detection were calculated to evaluate the diagnostic performance of the FF-QuantSC re-estimation workflow. The routine NIPT workflow had a sensitivity of 81.03%, specificity of 97.57%, PPV of 64.38%, and NPV of 98.96% for SCA detection. After FF-QuantSC recalibration, which successfully addressed the eight low-FF SCA cases, the performance improved to 94.83% sensitivity, 97.57% specificity, 67.90% PPV, and 99.71% NPV. Thus, the FF-QuantSC workflow demonstrated a marked increase in sensitivity.

### Analysis of Y-chromosome reads distribution

For 8 SCAs cases with “false-low” FF in the low-FF group (Table 2: case 1–8), we also performed an analysis of Y-chromosome reads distribution generated from NIPT data (Supplementary Figure S1). A consistent pattern was observed in all 8 cases, in which reads coverage across the entire Y-chromosome was slightly below the baseline, suggesting the possibility of a reduced copy number (low-level mosaic loss of the Y-chromosome). In 4 of these 8 cases (Cases 1, 3, 5, and 7), the reads distribution profiles also showed distinct regional deletions on the Y-chromosome. These observed patterns were also consistent with the SCA diagnoses obtained after FF-QuantSC recalibration in Table 4.

## Discussion

Our study demonstrates that low fetal fraction in NIPT is strongly associated with fetal Y-chromosome abnormalities. Crucially, our data suggest that Y chromosome-based FF estimation may underestimate FF in some cases with fetal Y-chromosome abnormalities, leading to “false-low” FF reports. This interpretation is supported by the observation that FF could be recalibrated to reportable levels using the Y-independent FF-QuantSC method in all eight such cases, enabling subsequent accurate NIPT risk calls for SCAs. The Y-chromosome reads distribution profiles also provide additional visual clues to indicate potential chromosomal abnormalities.

The strong independent association between low FF and SCAs in our cohort (OR = 78.227, 95% CI: 34.563–177.054, P < 0.001) presents a striking contrast to the established literature. A meta-analysis by Becking et al. demonstrated that low FF was associated with a higher risk of trisomy 13 (OR = 5.99), trisomy 18 (OR = 4.46), monosomy X (OR = 5.88), and triploidy (OR = 36.39), but not trisomy 21 (OR = 1.25) (Becking et al., 2023). The proposed explanation is that altered placental physiology, including a smaller placenta and a poorer maternal-fetal interface, leads to less cfDNA release into the maternal circulation, resulting in a lower FF. However, Lopes et al. and Caldwell et al. reported that although fetal trisomy may marginally correlate with reduced FF, no elevated trisomy rate was found in low FF failure cases (Lopes et al., 2020; Caldwell et al., 2021). Unlike these findings, our cohort revealed a distinct feature: among low-FF cases, there were no trisomies, but rather a dramatic enrichment of SCAs, specifically involving structural abnormalities or mosaicism loss of the Y-chromosome. This specificity suggests that the link between low FF and SCAs may be attributed to the integrity of the Y-chromosome. Of note, incomplete confirmatory testing may have overestimated the OR, given the much larger number of untested cases in the adequate-FF group. Nevertheless, the substantial difference in SCA prevalence between groups (8/70 vs. 50/24,020) is so large that even extreme assumptions would not negate a strong association.

Our data provide a further explanation for the observed association between Y-chromosome abnormalities and low FF. While we cannot fully exclude biological factors such as placental mosaicism, altered placental cfDNA shedding, or fetal-placental discordance, our findings support the possibility that the Y chromosome-based FF estimation method may underestimate FF in some cases with fetal Y-chromosome abnormalities. In our study, FF-QuantSC successfully recalculated FF to reportable levels (5.38%–19.90%) in all eight “false-low” cases, allowing subsequent NIPT analysis to correctly indicate high risk for SCAs (e.g., 45,X or Y deletions). Although the NIPT results were not completely consistent with the amniotic fluid karyotypes, this discrepancy can be explained by the fact that the proportion of mosaic cells may differ between the placenta and the fetus. This approach of FF recalibration offers a potential advantage over the standard protocol of sample redraw. Although FF is indeed positively correlated with gestational age (R^2^ = 0.27, P < 0.001, Figure 2), and the repeat draw with an average interval of 3.1 weeks successfully resolved 74.6% of initial low-FF cases in our study, this strategy is effective only for cases of truly low FF resulting from physiological factors. For “false-low” FF caused by Y-chromosome abnormalities, repeat testing typically fails to achieve a reportable FF (Table 4). In contrast, FF-QuantSC provides immediate correction, bypassing the need for redraw, and enabling timely and accurate detection of the underlying SCAs, with a marked improvement in sensitivity compared to routine NIPT. As for the two autosomal CNV cases in our low-FF group, they remained below the FF threshold after FF-QuantSC recalculation, confirming a “true” biologically low FF status.

Our findings are supported by other studies that have also questioned the accuracy of Y chromosome-based FF estimation. Zeng et al. reported two cases with a derivative Y-chromosome that could not be reliably detected using the Y chromosome-based method due to low FF; both cases showed adequate FF when reassessed using FF-QuantSC (Zeng et al., 2020). Xie et al. questioned the method’s accuracy after observing a male fetus predominance (80%) in their low-FF group and identifying two cases of Y-chromosome abnormalities within it (case 1: 45, X [10]/46, XY [41], case 2: Yq11.223-q11.23 deletion) (Xie et al., 2024). Persson et al. also pointed out that, due to imprecision in the FF estimation model, the vast majority of samples classified as no-call because their estimated FF fell below a cut-off would actually have a true FF above the cut-off (Persson and Prensky, 2021). These findings collectively demonstrate that fetal Y-chromosome abnormalities may interfere with the performance of the Y chromosome-based method for FF assessment, leading to “false-low” FF reports. FF-QuantSC can circumvent this limitation and accurately calculate FF because it relies on the differential genomic patterns between fetal and maternal genomes rather than on fetal sex chromosome (Yuan et al., 2020). This accurate assessment of sufficient FF enables NIPT to detect SCAs and other genome-wide chromosomal abnormalities, preventing missed detection. However, the error rate of FF prediction by FF-QuantSC is high at extreme FF values, which is possibly due to the limited number of samples with extreme FF in the training set, causing the artificial neural network to learn characteristics from samples with moderate FF. Thus, estimating the “true” FF can be challenging, we suggest that laboratories combine multiple FF evaluation methodologies to avoid algorithmic errors. Nevertheless, the Y-chromosome-based method remains the first-line FF estimation for male fetuses, with FF-QuantSC used only as a reflex test for low-FF cases or when a Y-chromosome abnormality is suspected.

Beyond FF recalculation, analysis of Y-chromosome read distribution profiles (Supplementary Figure S1) may provide additional visual clues. Although the eight SCA cases (Table 2: cases 1–8) yielded unreportable NIPT results due to falsely low FF, their Y-chromosome read distribution patterns showed features suggestive of possible underlying abnormalities (low-level mosaic loss or regional deletion). These observed patterns were also consistent with the SCA diagnoses obtained after FF-QuantSC recalibration in Table 4. This observation suggests that while the Y chromosome-based FF estimation algorithm may fail, the underlying sequencing data appear to retain biological information that can be interrogated through reads distribution analysis to reveal chromosomal anomalies. In cases with Y-chromosome deletions, such patterns were particularly noticeable. Although this analysis was not a definitive diagnostic tool, it may serve as a supportive clue that could prompt recalibration of FF before reporting a test failure, aiding in the detection of Y-chromosome abnormalities.

Our study has several limitations. First, confirmatory invasive testing was not performed uniformly in our cohort, which may have biased the true prevalence of SCAs and overestimated the observed association. Second, the number of SCA cases in the low-FF group was very small, limiting the precision of our estimates and the generalizability of the findings. Third, although our data suggest that the “false-low” FF in these cases is associated with the Y chromosome-based FF estimation method, biological contributions such as placental mosaicism, altered cfDNA shedding, or fetal-placental discordance cannot be fully excluded. Fourth, the mechanism by which only a subset of Y-abnormalities (66.7% in our cohort) triggers this “false-low” error remains unclear, and further investigation is complicated by confined placental mosaicism and the complex repeat structure of the Y chromosome. We identified a total of 12 cases of Y-chromosome abnormalities, with eight in the low-FF group (Table 2: cases 1–8) and the remaining 4 in the adequate-FF group (Table 3: cases 2, 3, 6, and 7). Fifth, our findings were obtained using a single sequencing platform (MGISEQ-2000) and a specific FF estimation algorithm (FF-QuantSC), and generalizability to other platforms or other FF estimation algorithms has not been established. Finally, as a single-center retrospective analysis, the proposed workflow needs prospective, multicenter studies to confirm its clinical utility.

In conclusion, our findings demonstrate that a “no-call” NIPT result due to low FF is strongly associated with fetal Y-chromosome abnormalities, suggesting that Y chromosome-based FF estimation may produce a “false-low” FF in some cases with fetal Y-chromosome abnormalities. Based on these observations, we suggest that a diagnostic workflow for low-FF NIPT samples, in which FF is re-estimated using a Y chromosome-independent method such as FF-QuantSC, complemented by assessment of Y-chromosome read distribution profiles, could be considered before classifying a sample as test failure. This workflow is promising but requires further prospective validation in larger cohorts before clinical routine implementation. If validated, it may enable earlier and more reliable identification of fetal Y-chromosome abnormalities, reduce unnecessary repeat blood draws, and shorten the time to definitive results.

## Data Availability

The original contributions presented in the study are included in the article/Supplementary Material, further inquiries can be directed to the corresponding authors.

## Supplementary material

SUPPLEMENTARY FIGURE S1 The Y-chromosome reads distribution profile of 8 SCAs cases in low fetal fraction group. The Y-chromosome read distribution profiles are generated from NIPT data analysis. The X-axis represents the continuous genomic position along the Y-chromosome, while the Y-axis indicates the Z-score of the normalized read depth per 50-kb bin. The baseline (Z = 0) corresponds to the expected read depth for a euploid region. In all eight cases, the profiles are consistently slightly below the baseline across the entire chromosome. Furthermore, a distinct regional deletion is identified on the Y-chromosome in case 1, case 3, case 5 and case 7.
