# Supplementary material for: Fetal Y chromosome abnormalities cause false-low fetal fraction in NIPT: a retrospective analysis of 24,101 pregnant women
Source: Front Genet. 2026 Jun 10;17:1856523. doi: 10.3389/fgene.2026.1856523 (PMC13290195; doi:10.3389/fgene.2026.1856523)
Supplement: Supplementary file 1 [file Table1.docx]

| **Supplementary Table 1. Ultrasound findings and pregnancy outcomes in 70 cases with low-FF.** | | | | | |  |  |  |
| --- | --- | --- | --- | --- | --- | --- | --- | --- |
| **Case** | **Mode of conception** | **Number of fetuses** | **Invasive test result** | **Ultrasound**  **findings** | **GA at delivery (weeks)** | **Neonatal sex** | **Birth weight (g)** | **Pregnancy outcome** |
| Case 1 | Natural | Singleton | SCA | Single umbilical artery | 39 | Male | 3190 | Live birth |
| Case 2 | Natural | Singleton | SCA | Hypospadias | 34 | Male | 2230 | TOP |
| Case 3 | IVF | Singleton | SCA | Normal | 39 | Male | 3020 | Live birth |
| Case 4 | Natural | Singleton | SCA | Normal | 28 | Male | 1300 | TOP |
| Case 5 | Natural | Singleton | SCA | Normal | 38 | Male | 3000 | Live birth |
| Case 6 | Natural | Singleton | SCA | Normal | 24 | Male | 680 | TOP |
| Case 7 | IVF | Twin | SCA | Fetus 1: Normal; Fetus 2: Normal | 24 | Fetus 1: Male; Fetus 2: Male | Fetus 1: 640; Fetus 2: 530 | TOP |
| Case 8 | Natural | Singleton | SCA | Normal | 39 | Male | 3240 | Live birth |
| Case 9 | IVF | Singleton | CNV | Normal | 31 | Male | 1710 | Live birth (preterm) |
| Case 10 | Natural | Singleton | CNV | Normal | 28 | Male | 1450 | TOP |
| Case 11 | Natural | Singleton | NA | Normal | 36 | Male | 1830 | Live birth (preterm) |
| Case 12 | Natural | Singleton | NA | Normal | 40 | Male | 3220 | Live birth |
| Case 13 | IVF | Singleton | NA | Normal | 36 | Female | 2730 | Live birth (preterm) |
| Case 14 | IVF | Twin | Normal | Fetus 1: Congenital heart disease; Fetus 2: Unilateral renal agenesis (left) | 37 | Fetus 1: Male; Fetus 2: Female | Fetus 1: 2440; Fetus 2: 2490 | Live birth |
| Case 15 | Natural | Singleton | NA | Normal | 36 | Male | 3030 | Live birth (preterm) |
| Case 16 | Natural | Singleton | NA | Normal | 41 | Male | 3280 | Live birth |
| Case 17 | Natural | Singleton | Normal | Normal | 39 | Male | 4010 | Live birth |
| Case 18 | Natural | Twin | Normal | Fetus 1: Congenital heart disease; Fetus 2: Congenital heart disease | 35 | Fetus 1: Male; Fetus 2: Female | Fetus 1: 1960; Fetus 2: 2130 | Live birth (preterm) |
| Case 19 | IVF | Singleton | Normal | Normal | 39 | Female | 3590 | Live birth |
| Case 20 | Natural | Singleton | NA | Normal | 40 | Male | 3110 | Live birth |
| Case 21 | Natural | Singleton | NA | Normal | 39 | Male | 2950 | Live birth |
| Case 22 | IVF | Singleton | NA | Normal | 35 | Female | 2380 | Live birth (preterm) |
| Case 23 | IVF | Singleton | NA | Normal | 39 | Female | 3265 | Live birth |
| Case 24 | Natural | Singleton | NA | Normal | 40 | Male | 3190 | Live birth |
| Case 25 | Natural | Singleton | NA | Normal | 40 | Male | 3825 | Live birth |
| Case 26 | Natural | Singleton | NA | Normal | 37 | Male | 3610 | Live birth |
| Case 27 | Natural | Singleton | NA | Normal | 34 | Male | 2195 | Live birth (preterm) |
| Case 28 | IVF | Singleton | NA | Normal | 38 | Male | 3815 | Live birth |
| Case 29 | Natural | Singleton | Normal | Normal | 38 | Male | 3850 | Live birth |
| Case 30 | Natural | Singleton | NA | Normal | 37 | Male | 2860 | Live birth |
| Case 31 | Natural | Singleton | Normal | Normal | 38 | Male | 2850 | Live birth |
| Case 32 | Natural | Singleton | NA | Normal | 39 | Male | 3165 | Live birth |
| Case 33 | Natural | Singleton | NA | Normal | 39 | Male | 2970 | Live birth |
| Case 34 | IVF | Twin | Normal | Normal | 36 | Fetus 1: Male; Fetus 2: Female | Fetus 1: 2600; Fetus 2: 2565 | Live birth (preterm) |
| Case 35 | IVF | Twin | NA | Normal | 37 | Fetus 1: Male; Fetus 2: Female | Fetus 1: 2750; Fetus 2: 2550 | Live birth |
| Case 36 | Natural | Singleton | NA | Normal | 36 | Male | 3360 | Live birth (preterm) |
| Case 37 | Natural | Singleton | NA | Normal | 37 | Male | 2560 | Live birth |
| Case 38 | IVF | Twin | Normal | Normal | 36 | Fetus 1: Male; Fetus 2: Female | Fetus 1: 2830; Fetus 2: 2350 | Live birth (preterm) |
| Case 39 | Natural | Singleton | NA | Normal | 38 | Female | 2820 | Live birth |
| Case 40 | Natural | Singleton | Normal | Normal | 41 | Female | 3370 | Live birth |
| Case 41 | Natural | Singleton | NA | Normal | 40 | Male | 3620 | Live birth |
| Case 42 | IVF | Singleton | Normal | Normal | 39 | Female | 3265 | Live birth |
| Case 43 | Natural | Singleton | Normal | Normal | 38 | Female | 3010 | Live birth |
| Case 44 | Natural | Singleton | NA | Normal | 40 | Female | 3200 | Live birth |
| Case 45 | Natural | Singleton | Normal | Bilateral mild pyelectasis | 37 | Male | 3000 | Live birth |
| Case 46 | IVF | Singleton | Normal | Placenta previa | 37 | Female | 2560 | Live birth |
| Case 47 | Natural | Singleton | Normal | Normal | 30 | Male | 1620 | Live birth (preterm) |
| Case 48 | Natural | Singleton | NA | Normal | 31 | Male | 1380 | Live birth (preterm) |
| Case 49 | IVF | Singleton | Normal | Normal | 39 | Female | 3160 | Live birth |
| Case 50 | IVF | Singleton | Normal | Normal | 38 | Female | 3045 | Live birth |
| Case 51 | IVF | Singleton | NA | Velamentous cord insertion | 38 | Female | 2860 | Live birth |
| Case 52 | IVF | Singleton | Normal | Normal | 36 | Female | 3150 | Live birth (preterm) |
| Case 53 | IVF | Singleton | Normal | Normal | 39 | Female | 3090 | Live birth |
| Case 54 | Natural | Singleton | Normal | Normal | 41 | Female | 3440 | Live birth |
| Case 55 | Natural | Singleton | NA | NA | 16 | Female | NA | Spontaneous abortion |
| Case 56 | IVF | Singleton | NA | Normal | NA | NA | NA | NA |
| Case 57 | IVF | Singleton | NA | NA | NA | NA | NA | NA |
| Case 58 | Natural | Twin | NA | NA | NA | NA | NA | NA |
| Case 59 | Natural | Twin | NA | NA | NA | NA | NA | NA |
| Case 60-70 | Natural | Singleton | NA | NA | NA | NA | NA | NA |

Cases 1–10 in this table correspond to the same cases as presented in Table 2. IVF, in vitro fertilization. SCA, sex chromosome abnormality. CNV, copy number variation. GA, gestational age. TOP, termination of pregnancy. NA, not available.
