# Supplementary figures and images for: Fetal Y chromosome abnormalities cause false-low fetal fraction in NIPT: a retrospective analysis of 24,101 pregnant women
Source: Front Genet. 2026 Jun 10;17:1856523. doi: 10.3389/fgene.2026.1856523 (PMC13290195; doi:10.3389/fgene.2026.1856523)

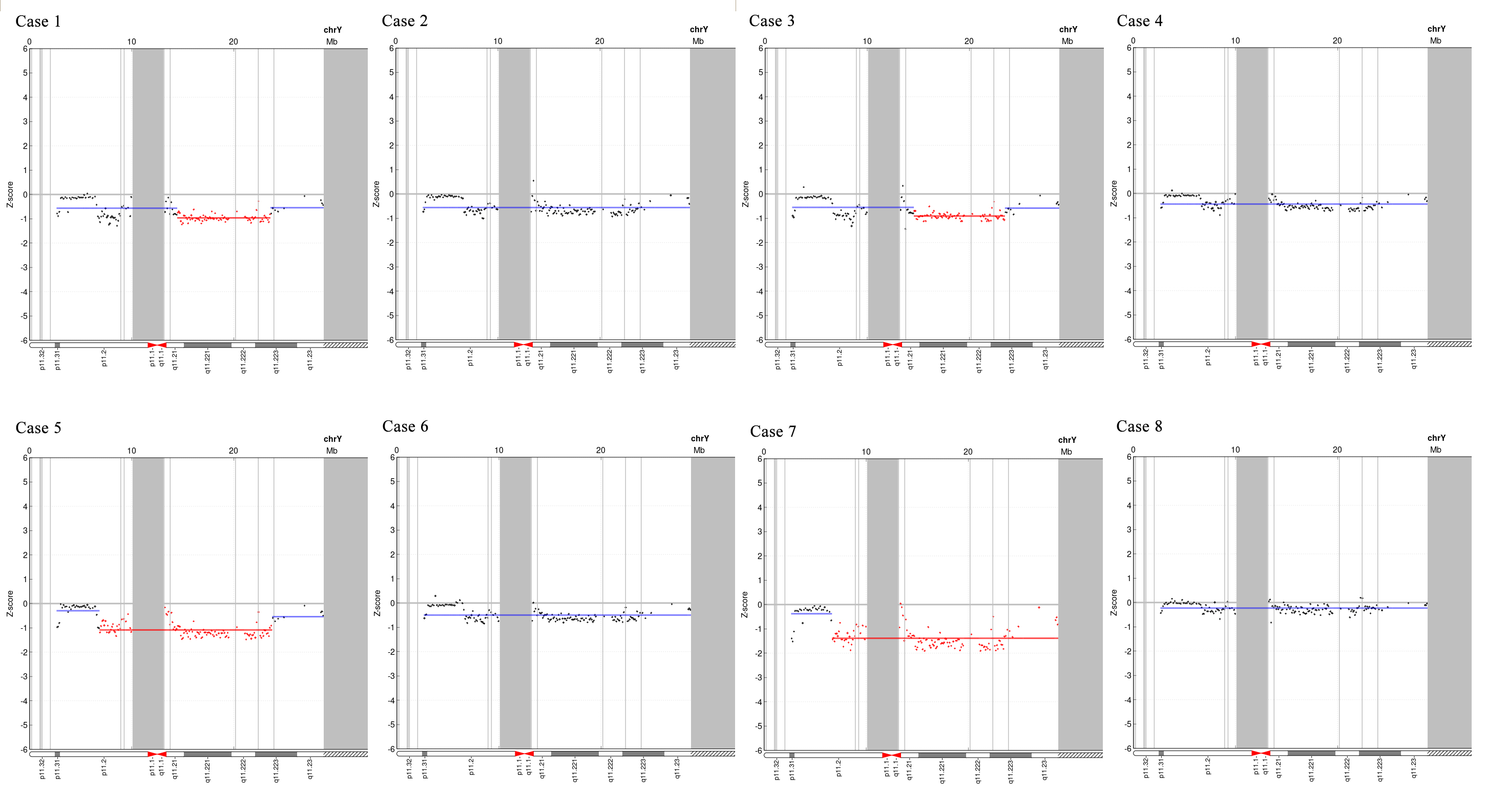

Supplement: Supplementary file 2 [file Image1.jpeg]
