# Supplementary material for: Fetal Y chromosome abnormalities cause false-low fetal fraction in NIPT: a retrospective analysis of 24,101 pregnant women
Source: Front Genet. 2026 Jun 10;17:1856523. doi: 10.3389/fgene.2026.1856523 (PMC13290195; doi:10.3389/fgene.2026.1856523)
Supplement: Supplementary file 3 [file Table2.docx]

| **Supplementary Table 2. Abbreviation list.** | |
| --- | --- |
| **Abbreviation** | **Full Term** |
| BMI | body mass index |
| CI | confidence intervals |
| CMA | chromosome microarray analysis |
| CNVs | copy number variations |
| cfDNA | cell-free DNA |
| FF | fetal fraction |
| FF-QuantSC | Quantification of FF with Shallow-Coverage sequencing |
| IVF | in vitro fertilization |
| MCA | multiple chromosomal aberrations |
| NPV | negative predictive value |
| NIPT | non‑invasive prenatal testing |
| OR | odds ratio |
| PPV | positive predictive value |
| QC | quality control |
| RATs | rare autosomal trisomies |
| SCAs | sex chromosome abnormalities |
| SD | standard deviation |
